# The treatment of Wilms' tumour: results of the United Kingdom Children's Cancer Study Group (UKCCSG) second Wilms' tumour study

**DOI:** 10.1054/bjoc.2000.1338

**Published:** 2000-08-16

**Authors:** C Mitchell, P Morris Jones, A Kelsey, G M Vujanic, B Marsden, R Shannon, P Gornall, C Owens, R Taylor, J Imeson, H Middleton, J Pritchard

**Affiliations:** Paediatric Oncology, Oxford Radcliffe Hospital, Oxford, OX 9DU; Royal Manchester Children’s Hospital, Pendlebury, Manchester, M27 1HA; Department of Histopathology, University of Wales College of Medicine, Cardiff, CF4 4XN; Paediatric Oncology, Leicester Royal Infirmary, Leicester, LE1 5WW; Department of Surgery, Birmingham Children’s Hospital, Birmingham, B4 6NH; Department of Radiology, Great Ormond Street Hospital for Children, London, WC1N 3JH; Cookridge Hospital, Leeds, LS16 6QB; UKCCSG Data Centre, University of Leicester, Leicester, LE1 6TP; Institute of Child Health, Guilford Street, London, WC1 N3 JH, UK

**Keywords:** Wilms’, tumour, treatment, chemotherapy, radiotherapy, childhood cancer

## Abstract

The aims of the UKW2 study were: (1) to further refine treatment for stage I and II favourable histology (FH) patients; (2) to consolidate the UKW1 results for stage III FH patients; (3) to improve the outlook for patients with inoperable primary tumours and those patients with stage IV and unfavourable histology disease. Treatment consisted of primary nephrectomy, wherever possible, followed by chemotherapy and radiotherapy, as dictated by stage and histology. Treatment was refined successfully for stage I and II FH patients. The 4-year event-free survival for these two groups was 94% and 91%, respectively. Stage III FH patients had a 4-year event free survival of 84%. The outlook for patients with clear cell sarcoma of the kidney is as good as for patients with favourable histology, whilst that for patients with anaplastic or rhabdoid variants remains poor. The outlook for the majority of children with Wilms’ tumour is now excellent. © 2000 Cancer Research Campaign


*British Journal of Cancer* (2000) **83**(5), 602–608
© 2000 Cancer Research Campaign
doi: 10.1054/ bjoc.2000.1338, available online at http://www.idealibrary.com on IDEAL®

### The treatment of Wilms' tumour: results of the United Kingdom Children's Cancer Study Group (UKCCSG) second Wilms' tumour study

**C Mitchell[1], P Morris Jones[2], A Kelsey[3], GM Vujanic[3], B Marsden[2], R Shannon[4], P Gornall[5], C Owens[6], R Taylor[7], J Imeson[8], H Middleton[8] and J Pritchard[9] for the UKCCSG**

[1]Paediatric Oncology, Oxford Radcliffe Hospital, Oxford OX 9DU; [2]Royal Manchester Children's Hospital, Pendlebury, Manchester M27 1HA; [3]Department of Histopathology, University of Wales College of Medicine, Cardiff CF4 4XN; [4]Paediatric Oncology, Leicester Royal Infirmary, Leicester LE1 5WW; [5]Department of Surgery, Birmingham Children's Hospital, Birmingham B4 6NH; [6]Department of Radiology, Great Ormond Street Hospital for Children, London WC1N 3JH; [7]Cookridge Hospital, Leeds LS16 6QB; [8]UKCCSG Data Centre, University of Leicester, Leicester LE1 6TP; [9]Institute of Child Health, Guilford Street, London WC1 N3 JH, UK

**Summary** The aims of the UKW2 study were: (1) to further refine treatment for stage I and II favourable histology (FH) patients; (2) to consolidate the UKW1 results for stage III FH patients; (3) to improve the outlook for patients with inoperable primary tumours and those patients with stage IV and unfavourable histology disease. Treatment consisted of primary nephrectomy, wherever possible, followed by chemotherapy and radiotherapy, as dictated by stage and histology. Treatment was refined successfully for stage I and II FH patients. The 4-year event-free survival for these two groups was 94% and 91%, respectively. Stage III FH patients had a 4-year event free survival of 84%. The outlook for patients with clear cell sarcoma of the kidney is as good as for patients with favourable histology, whilst that for patients with anaplastic or rhabdoid variants remains poor. The outlook for the majority of children with Wilms' tumour is now excellent. © 2000 Cancer Research Campaign

**Keywords**: Wilms' tumour; treatment; chemotherapy; radiotherapy; childhood cancer

Although the prognosis for children with Wilms' tumour improved steadily from the 1960s until the early 1980s, there has been relatively little progress, so far as treatment intensity is concerned, since then. With overall long-term survival now exceeding 80%, investigators have instead invested their efforts into planned reduction of both short- and long-term toxicities, especially in those patients – fortunately the majority – who have biologically favourable disease. The second national United Kingdom Children's Cancer Study Group (UKCCSG) Wilms' tumour study (UKW2), reported here, is an example of this genre of studies.

The Medical Research Council, who conducted two trials, MRC1 from 1970–1974 (Lennox et al, 1979) and MRC2 from 1974–1978; (Morris Jones et al, 1987) initiated national treatment studies for Wilms' tumour in the UK. MRC1 and MRC2 enrolled 108 and 144 patients respectively, aged over 1 year with non-metastatic disease, all of whom were treated with nephrectomy, abdominal radiotherpay and then chemotherapy. More than 70% of patients in MRC1 were still alive at 3 years, an encouraging result for that era. The 62 stage 1 patients in MRC2 were randomized to receive either 6 months or 2 years of single-agent actinomycin D. The outcome for the two groups was identical with a 3-year survival of 86%. The 82 stage II and III patients were randomized to either a 2-drug regimen (vincristine and actinomycin D) or 3-drug regimen (these two drugs plus doxorubicin) after nephrectomy and postoperative radiotherapy. The outcome for the two groups was identical.

The results for stage I patients in MRC2 conflicted with an earlier US study in which there appeared to be an advantage to a longer course of treatment, albeit at a lower dose intensity (D'Angio et al, 1976). The stage II and III results also conflicted with the findings of a similar randomization in the much larger National Wilms' Tumour Study (NWTS) 2 trial, which demonstrated an advantage for patients receiving a similar 3-drug regimen (D'Angio et al, 1981).

The UKCCSG assumed responsibility for UK national Wilms' tumour trials in 1979. Its first study, 'UKW1', included all patients with all stages and histological subtypes of Wilms' tumour. Children with metastatic disease at diagnosis (stage IV) and those with clear cell sarcoma (CCSK) and rhabdoid tumours (RTK) were also included (Pritchard et al, 1995). The main objective of UKW1 for favourable histology (FH) patients was to determine whether treatment could be reduced in stages I and II patients without compromising cure rates, and whether intensification of therapy for stage III and IV and unfavourable histology (UH) patients could improve prognosis. This study demonstrated that for stage 1 FH patients, single-agent vincristine was as effective as vincristine and actinomycin D, and that fractionation of actinomycin D was unnecessary. The results for children with stage III tumours were comparable to those of contemporary NWTS trials, but those for stage IV and UH Wilms' patients seemed to be inferior to those of the NWTS studies, either because of differences in treatment or in case selection bias or both. The results for children with CCSK and RTK were similar to those achieved by the NWTS.

*Received 18 November 1999*
*Revised 4 May 2000*
*Accepted 17 May 2000*

*Correspondence to:* C Mitchell

The aims of UKW2 were to:

1. explore the possibility of further refinements in treatment for stage I and II FH patients, by reducing the duration of single-agent vincristine treatment for stage I patients from 6 months to 10 weeks and by omitting radiotherapy for stage II patients
2. consolidate the UKW1 results for stage III FH patients who received the same treatment as in UKW1 (Pritchard et al, 1995)
3. improve the outlook for patients with inoperable primary tumours and those patients with stage IV and UH disease, including RTK and CCSK, by intensification of chemotherapy and addition of radiotherapy.

### METHODS

### Patient eligibility, histology and staging

All the then-20 constituent centres of the UKCCSG (see Appendix) participated in the study from its inception in July 1986 until its closure in September 1991. In all cases the diagnosis of Wilms' or other renal tumour was confirmed histopathologically, either from the nephrectomy specimen or, in those patients whose tumours were deemed inoperable, by open or closed needle biopsy. All UK-based children with newly diagnosed Wilms' tumour diagnosed prior to their 15th birthday were eligible, including patients with metastatic or bilateral tumours. The only exclusions were those patients who had previously received treatment for Wilms' tumour.

Nephrectomy was the initial treatment in all patients except those with stage IV or inoperable tumours. The 'local' pathologist initially determined the abdominal stage and histology of each tumour. Multiple blocks were taken from each tumour (a minimum of ten was recommended) around its maximum circumference, as well as from areas where there was suspicion of tumour spread beyond the pseudocapsule. All excised lymph nodes were examined. Subsequently, histopathological sections were reviewed by a panel of pathologists and classified as Wilms' tumour (WT) favourable histology (FH); anaplastic WT (focal or diffuse); rhabdoid tumour (RTK), clear cell sarcoma (CCSK or bone-metastasizing renal tumour), mesoblastic nephroma, renal cell carcinoma or primitive neuroectodermal tumour. The staging system and histological subtyping were those used in UKW1 and correspond to those used in the NWTS 3 and NWTS 4 studies (D'Angio et al, 1989; Green et al, 1995).

Other investigations for all patients included abdominal ultrasound or CT scan, and postero-anterior plus lateral chest X-rays (CXR). CT scans of the chest were performed in a number of patients, but metastases visible on plain CXR were required for patients to be classified as stage IV. Isotopic or radiological skeletal surveys were required only in those patients with CCSK. Cranial CT scans were recommended for patients with rhabdoid tumours. Renal angiography and intravenous urogram were optional. Based on the staging investigations, the surgical and pathological findings and the criteria established by the NWTS, patients were classified as having either FH or UH tumours, stages I, II, III or IV, or bilateral primary tumours. 'Inoperable' non-metastatic tumours were considered to be stage III.

### Treatment plan

All patients without metastases at diagnosis were, if the tumour was deemed operable by the local surgeon, to have initial surgery. All patients received chemotherapy according to their surgical stage. In addition all stage III and IV patients were to receive radiotherapy to the tumour bed and/or sites of abdominal or lung metastases. The overall treatment strategy and details of the chemotherapy are shown in Table 1.

**Table 1** Overall treatment plan and details of chemotherapy

| Histology/stage | Surgery | Radiotherapy | Chemotherapy |
|---|---|---|---|
| Favourable histology | | | |
| I | Initial | None | VCR only |
| II | Initial | None | VCR + ActD |
| III | Initial | 20 Gy, hemi-abdomen | Non-intensive VCR + ActD + doxo |
| IV | Delayed | If local stage III 30 Gy to hemi-abdomen; 12 Gy to whole lung | Intensive VCR + ActD + doxo |
| Unfavourable histology | | | |
| I | Initial | None | Intensive VCR + ActD + doxo |
| II | Initial | None | Intensive VCR + ActD + doxo |
| III | Initial | 30 Gy to hemiabdomen | Intensive VCR + ActD + doxo |
| IV | Delayed | If local stage III 30 Gy to hemi-abdomen; 12 Gy to whole lung | Intensive VCR + ActD + doxo |
| Inoperable tumours, any histology | Delayed | 30 Gy to hemi-abdomen, if local stage III at time of surgery | Intensive VCR + ActD + doxo |

**FH Stage I:** 10 weekly injections of VCR 1.5 mg $m^{-2}$ as a single agent.
**FH Stage II:** 11 weekly injections of VCR, then 3-weekly for five further doses, together with ActD every 3 weeks starting at week 2 for a total of nine doses. Total duration of treatment, 6 months.
**FH Stage III:** 10 weekly doses of VCR, followed by 14 further doses at 3-weekly intervals; ActD 0.75 mg $m^{-1}$ at week 2 just prior to radiotherapy and then 1.5 mg $m^{-2}$ at week 10 then at 6-weekly intervals until week 52; doxo 40 mg $m^{-1}$ at 6-weekly intervals alternating with ActD, starting at week 7. Total duration of treatment, 12 months.
**FH Stage IV:** 11 weekly injections of VCR, then 3-weekly for five further doses, together with ActD every 3 weeks starting at week 2 for a total of nine doses concurrently with doxo 30 mg $m^{-1}$. Total duration of treatment, 6 months.
**UH Stage III and primarily inoperable tumours:** as for UH stages I and II. Total duration of treatment, 12 months.

VCR = vincristine; ActD = actinomycin D; doxo = doxorubicin

© 2000 Cancer Research Campaign

### Surgery

Initial surgery, i.e. surgery at the time of diagnosis, was recommended for all patients unless the operation was deemed too risky, or complete tumour excision could probably not be achieved. In this case, and for patients with metastases detected at the time of diagnosis, patients received three-drug chemotherapy and had a delayed nephrectomy at approximately week six. Recommendations for surgery were identical to those used in UKW1 (Pritchard et al, 1995).

### Chemotherapy

Details of chemotherapy are given in Table 1.

### Radiotherapy

*Abdomen*

Patients with operable FH stage III tumours were to be given 20 Gy (10 × 2 Gy) to the midplane of the tumour over 2 weeks starting within 14 days of surgery. Patients with delayed surgery did not receive any radiotherapy if there was no viable tumour at the time of surgery but received 30 Gy (15 × 2 Gy) to the flank if viable tumour was present.

*Lung*

Whole-lung irradiation was to be given to all patients with metastases present on chest X-ray at the time of diagnosis. Radiotherapy was given immediately after surgery and concurrently with abdominal radiotherapy if also indicated. Treatment consisted of 12 Gy to the midplane of the lungs, given as 8 × 1.5 Gy fractions.

### Data collection and analysis

Data on all patients with renal tumours treated in UKCCSG centres, representing more than 90% of all childhood renal tumours diagnosed in the UK during the 63-month period of UKW2 (Stiller et al, unpublished), was collected centrally and reviewed by the study coordinators. Survivals were calculated by the method of Kaplan and Meier (1958). The log-rank test (Peto et al, 1977) was applied to evaluate the significance of established prognostic factors – stage, histological subtype and age-group (< 1 year, 1–4 years and > 5 years). Survival time was defined as the time from diagnosis to death from any cause, or to date of last follow-up. Event-free survival was defined as the time from diagnosis to first relapse/progression, time to death or date of last follow-up.

The local centre determined the tumour stage according to NWTS criteria. Subsequent central review was also carried out. Assessment of agreement between 'local' and 'central' review panels used the kappa statistic (Brennan and Silman, 1992).

### RESULTS

Between June 1986 and September 1991, UKCCSG centres registered a total of 447 consecutively diagnosed eligible patients with renal tumours. Three patients with extra-renal tumours were included but not allocated a stage. Analysis followed the 'intention to treat' principles, i.e. patients with protocol deviations were included. Brief details of the patients with unfavourable histology are given here, but will be more comprehensively reported by Kelsey et al (in preparation).

The male:female ratio was 207:240 (0.86:1) and the age-range at diagnosis was 0–15.4 years (median 2.71 years). Of the 46 UH patients, 20 were boys. No gender preponderance was noted in the group with CCSK (8 boys, 8 girls).

Twenty UKCCSG centres entered patients in the study, median 18 patients, range 1–100 (see Appendix). One centre entered a single patient, 12 centres entered fewer than 20 patients and eight centres 20 patients or more. There was no indication of any difference between the large and small centres in overall survival (OS) ($P = 0.86$) or event-free survival (EFS) ($P = 0.59$). Outcomes were also similar when only Wilms' patients were considered, with $P$-values of 0.77 and 0.30 for OS and EFS, respectively.

### Stage

The stage distribution of patients with favourable and other histologies is given in Tables 2 and 3 respectively. The three patients with extra-renal tumours were not allocated a stage and so cannot be included in the stage-related analysis. There were 23 patients with bilateral tumours who are included in the analysis, but have been more fully described previously (Kumar et al, 1998).

### Survival

Patients have been followed-up to April 1998, a median of 103 months (range 3–139 months) since diagnosis. There have been 70 deaths in all, 63 from tumour, three from treatment complications and four from other causes. The 2-year and 4-year estimates of overall and event-free survival by stage for 398 FH patients are in Table 4. As expected, stage is significantly correlated with both EFS and OS with log-rank test $P$-values for differences in survival between stages of $P = 0.05$ for EFS and $P = 0.0008$ for OS.

### Histology

According to the local pathologists' reports, there were 401 favourable histology (FH) patients and 46 unfavourable histology (UH) patients (17 clear cell sarcoma (BMRTC), 20 anaplastic, nine rhabdoid). In just one patient the histology showed Wilms' tumour but the subtype could not be determined.

**Table 2** Stage distribution for 398 favourable local histology patients

| Stage | *n* (%) |
|---|---|
| I | 136 (34) |
| II | 57 (14) |
| III[a] | 122 (31) |
| IV | 60 (15) |
| V | 23 (6) |

[a]includes 36 tumours initially unresectable

**Table 3** Stage distribution for 46 unfavourable local histology patients

| Stage | Anaplasia | CCSK | RTK |
|---|---|---|---|
| I | 6 | 7 | 0 |
| II | 3 | 4 | 1 |
| III | 5 | 6 | 6 |
| IV | 6 | 0 | 2 |

© 2000 Cancer Research Campaign

**Table 4** 2-year and 4-year estimates of overall survival and event-free survival for 398 favourable histology patients, stratified according to local histology and staging

| Stage | *n* (%) | 2-year OS (% [95% CI]) | 2-year EFS (% [95% CI]) | 4-year OS (% [95% CI]) | 4-year EFS (% [95% CI]) |
|---|---|---|---|---|---|
| I | 136 (34) | 96 [91–98] | 89 [83–93] | 94 [89–97] | 87 [80–91] |
| II | 57 (14) | 95 [85–98] | 82 [72–92] | 91 [81–96] | 82 [70–90] |
| III | 122 (31) | 90 [84–94] | 83 [75–88] | 84 [77–90] | 82 [74–88] |
| IV | 60 (15) | 82 [70–89] | 72 [59–82] | 75 [63–84] | 70 [57–80] |
| Bilateral | 23 (6) | 83 [63–93] | 70 [49–84] | 78 [58–90] | 70 [49–84] |

**Table 5** 2-year and 4-year overall survival and event-free survival by 'review' histology

| Review histology | 2-year OS *n* (%) | 2-year EFS (% [95% CI]) | 4-year OS (% [95% CI]) | 4-year EFS (% [95% CI]) | (% [95% CI]) |
|---|---|---|---|---|---|
| All favourable histology | 338 | 91 [87–93] | 83 [79–87] | 87 [83–90] | 82 [77–86] |
| Clear cell sarcoma | 18 | 88 [66–97] | 82 [59–94] | 88 [66–97] | 82 [59–94] |
| Anaplastic | 14 | 64 [39–84] | 36 [16–61] | 50 [27–73] | 29 [11–56] |
| Rhabdoid | 11 | 36 [15–65] | 36 [15–65] | 36 [15–65] | 36 [15–65] |

**Table 6** Comparison between 'local' and 'review' tumour stage for 374 patients

| | Review stage | | | | | |
|---|---|---|---|---|---|---|
| Local stage | I | II | III | IV | Bilateral | Kappa |
| I | 124 | 4 | 1 | 0 | 0 | 0.90 |
| II | 6 | 38 | 11 | 0 | 0 | 0.65 |
| III | 2 | 9 | 105 | 0 | 0 | 0.81 |
| IV | 3 | 3 | 8 | 45 | 0 | 0.84 |
| Bilateral | 1 | 0 | 0 | 0 | 14 | 0.96 |
| Overall Kappa | – | – | – | – | – | 0.82 |

Histology, as assessed both locally and by the review panel, significantly correlated with differences in outcome. Patients with FH and CCSK fared better than patients with anaplastic tumours and RTK (91 and 94% vs 55 and 33% respectively). For all 447 patients, the log-rank test for OS gave $P < 0.0001$, and for EFS $P < 0.0001$ for differences in outcome. Details of outcome by review pathology are given in Table 5.

### Age and gender

Age did not affect outcome. For the 401 FH patients, the log-rank test for differences in outcome by age gave for overall survival (OS) $P = 0.69$ (Trend test 0.45), and for event-free survival (EFS) $P = 0.81$ (Trend test 0.97), (data not shown). Overall, girls fared slightly less well than boys but the difference was not statistically significant (data not shown). In multivariate analysis of all 447 patients, after adjustment of outcome for stage and histology, the difference by gender is not significant for EFS ($P = 0.12$).

### Stage

Three hundred and seventy four patients' records were retrospectively examined by central review. Review stage agreed with the local assessment in 326 cases (87%), but differed in 48. The major disagreement concerned the allocation to state II or III; some patients had been mis-assigned a stage despite clear pathological reports to the contrary. In some instances the pathologist had indicated an incorrect stage, while in others the treating oncologist had drawn an erroneous conclusion from the pathologist's report. Some patients initially assigned to stage IV were reassigned to stages I–III because there was no record of metastatic deposits either on plain chest radiographs or at laparotomy. Two patients were assigned to stage IV because of lung metastases detected solely on CT scan (Owens et al, in preparation).

Kappa analysis (Brennan and Silman, 1992) evaluates how much better agreement between local and review assessments are than chance. Conventionally, a value above 0.8 is taken as 'very good' agreement, and above 0.6 as 'good' agreement. Overall there is very good agreement particularly for stage I and bilateral tumours. Eleven of 55 stage II patients (local assessment) should have been upstaged to III, but only 11/116 patients were mistakenly upstaged locally from stage I or II to stage III locally. Details of this analysis are shown in Table 6.

### Histology

Material from 381 patients was available for central review. In 366 cases review histology agreed with the local assessment (96%). According to the pathology review panel, there were 43 unfavourable histology (UH) cases (14 anaplastic Wilms', 18

*© 2000 Cancer Research Campaign*

CCSK, 11 RTK), 338 favourable histology (FH) and 67 patients with insufficient material for definitive conclusions. For all reviewed patients, differences in outcome by review histology were highly significant (log-rank test: OS $P < 0.0001$, and EFS $P < 0.0001$).

By kappa analysis there was very good overall agreement between local and review assessment, particularly for clear cell sarcoma and rhabdoid tumours. Five (30%) of the 16 tumours designated by the local pathologist as anaplastic were regarded as FH by the review panel, whilse seven of 339 tumours identified locally as FH were classified as UH on review. Nevertheless, the survival outcome for all histological subtypes is similar whether analysed by local or central review.

### DISCUSSION

Investigators agree that outcome is likely to be good for Wilms' patients with no unfavourable histological features and no imaging evidence of metastasis – in other words those with stage I, II and III FH tumours (Green et al, 1996*a*; Godzinski et al, 1999). Children with stage I tumours with focal anaplasia and, so long as doxorubicin is included in the treatment regimen, CCSK, also have a good prognosis. Patients with FH tumours but radiological (CXR) evidence of metastatic disease in the lungs or elsewhere (stage IV) have a moderate outlook, whereas those with stages II, III and IV RTK have a poor prognosis. Recent Wilms' tumour trials have shared objectives – to reduce toxicity, especially 'late effects' of treatment, in patients with a relatively good prognosis, and to improve survival particularly in those with a less favourable prognosis. The UKW2 trial was no exception. Comparisons with NWTS trials are straightforward, given the similarities in approach. Comparisons with SIOP trials, though, are made difficult by differences in patient stratification and the use of preoperative chemotherapy.

The results of UKW2 show, as do UKW1, NWTS 1, 2, 3 and 4 and recent SIOP studies, that treatment for patients with good prognosis can be successfully refined without prejudicing survival, or even the rate of relapse (D'Angio et al, 1976; 1981; 1989; Tournade et al, 1993; Green and Coppes 1995; Pritchard et al, 1995; Godzinski et al, 1999). About 90% of children with stage I FH tumours (34% of all FH patients in UKW2) can be cured by radical tumour removal and single-agent chemotherapy with only 10 weekly doses of vincristine. Results in UKW2 are virtually the same as those obtained in the contemporary NWTS3: 4-year EFS 87 vs 91.8%, and 4-year OS 94 vs 97.4% using 6 months of vincristine and actinomycin D. Thus, in these patients, the risk of actinomycin D-induced hepatotoxicity (Green et al, 1988; Raine et al, 1991; Ludwig et al 1992), which can be life-threatening, can be avoided, as can myelosuppression with its attendant risks, and alopecia.

More than 90% of stage II FH patients can also be cured with 6 months of two-drug treatment using vincristine and actinomycin D, without exposing them to the risks of treatment with radiotherapy or doxorubicin. Similar results were seen in NWTS3, where 'intensive' actinomycin D with vincristine was seen to be as good as three-drug chemotherapy, and the addition of 20 Gy radiotherapy did not significantly improve outcome. In both stage I and stage II patients, therefore, the objectives of UKW2 were achieved.

Stage III patients in UKW2 were treated in exactly the same manner as UKW1, with three-drug chemotherapy (vincristine, actinomycin D and doxorubicin) and 20 Gy abdominal radiotherapy, usually to the hemiabdomen. In UKW1, subset analysis suggested that patients with tumours designated 'stage III' only by virtue of microscopic tumour at resection margins fared better than those with visible tumour residue or positive abdominal lymph nodes or both (UKCCSG, unpublished observation). This observation led one UKCCSG centre to conduct, during UKW2, a separate pilot study in which radiotherapy was omitted successfully from the treatment of its stage III patients < 3 years-of-age at diagnosis, judged as particularly vulnerable to troublesome 'late effects' from radiotherapy (Pachnis et al, 1998). The results of our analysis of stage III results from UKW2 will be published separately.

Survival for stage IV FH patients was better in UKW2 than in UKW1, but the comparison is historical and there may be a number of explanations for the difference. The growing overall experience of Wilms' tumour management in UKCCSG centres might, for instance, be critical, as might the inclusion of whole-lung irradiation in children with lung metastases. Even though the treatment plan was similar, UKW results for stage IV patients are apparently inferior to those reported in NWTS studies (D'Angio et al, 1989; Pritchard et al, 1995; Green et al, 1996*b*). However, of the 59 patients assessed locally as stage IV, only 37 had whole-lung radiotherapy as prescribed by the protocol. Superficially, there is a clear case for whole-lung radiotherapy in the treatment of lung metastases in Wilms' tumour. The SIOP group, however, have reported survival of over 80% in stage IV patients without systematic use of pulmonary radiotherapy (De Kraker et al, 1990), and 50% of stage IV patients in UKW1 were cured without its use (Pritchard et al, 1995). It is possible that with careful selection of patients and greater use of pulmonary resection, the proportion of patients receiving pulmonary radiotherapy might be reduced without prejudicing their chances of survival, but whether such a development is warranted, given the minimal long-term consequences of 12 Gy of pulmonary radiotherapy, is questionable. A more relevant question might be the identification of patients who could be successfully treated with less doxorubicin, thus obviating the potential long-term side-effects of anthracycline therapy, particularly when given in conjunction with whole-lung radiotherapy (Lipshultz et al, 1991; Sorensen et al, 1995). The NWTS concluded that there was a subgroup of stage IV patients who could be successfully treated without doxorubicin (Green et al, 1996*b*). The NWTS have also now demonstrated that treatment for stage III and IV tumours can be shortened, and hence the cumulative dose of doxorubicin reduced, without prejudicing outcome (Green et al, 1998).

Except for CCSK patients, whose cure rate overall is now as good that for FH patients, results for children with unfavourable histology renal tumours are still disappointing. Patients with anaplastic tumours have a 4-year EFS of around only 30%. Data from NWTS3 suggested that stage II–IV patients with anaplasia might benefit from the addition of cyclophosphamide, although this difference was not significant once allowance had been made for the number of patients with only focal anaplasia receiving this additional drug. Children with RTK have an appalling prognosis unless they have a stage I tumour. Better treatments are urgently needed for these patients.

The histology and stage review in this study was carried out after it had closed, and for the purposes of verifying the accrued data. It was not intended as a method of ensuring rigorous

© 2000 Cancer Research Campaign adherence to the protocol. Nevertheless, the differences in 'local' and 'review' staging emphasize the need for particular care in this process. Patients treated for a lower-stage tumour than they actually have will have an increased risk of recurrence. In this study, 16 of 374 patients were understaged, of whom 12 ought to have been stage III and who therefore did not receive have doxorubicin and flank irradiation. Conversely, 29 of 374 patients were overstaged, of whom 24 received doxorubicin and radiotherapy with their attendant long-term toxicities. Thus 12% of patients overall had incorrect staging. Histology was more accurate, with local and review opinions differing in only 14 (3%) out of 380 cases. Future studies probably ought to include some method for rapid review of pathology and stage to ensure the highest standards of protocol compliance.

### ACKNOWLEDGEMENTS

This study was supported by a grant to the UKCCSG from the Cancer Research Campaign.

### REFERENCES

Brennan P and Silman A (1992) Statistical methods for assessing observer variability in clinical measures. *BMJ* **304**: 1491–1494

D'Angio GJ, Evans AE, Breslow N, Beckwith B, Bishop H, Feigl P, Goodwin W, Leape LL, Sinks LF, Sutow W, Tefft M and Wolff J (1976) The treatment of Wilms' tumour. Results of the first National Wilms' tumour study. *Cancer* **38**: 633–646

D'Angio GJ, Evans AE, Breslow N, Beckwith B, Bishop H, Farewell V, Goodwin W, Leape L, Palmer N, Sinks L, Sutow W, Tefft M and Wolff J (1981) The treatment of Wilms' tumour: results of the second National Wilms' tumour study. *Cancer* **47**: 2302–2311

D'Angio GJ, Breslow N, Beckwith JB, Evans AE, Baum H, de Lorimier A, Fernbach D, Hrabovsky E, Jones B and Kelalis P (1989) The treatment of Wilms' tumour. Results of the third National Wilms' tumour study. *Cancer* **64**: 349–360

De Kraker J, Lemerle J, Voute PA, Zucker JM, Tournade MF and Carli M (1990) Wilms' tumour with pulmonary metastases at diagnosis: The significance of primary chemotherapy. *J Clin Oncol* **8**: 1187–1190

Godzinski J, Tournade MF, De Kraker J, Ludwig R, Weirich A, Voute PA, Burgers JM, Habrand JL, Sandstedt B and Ducourtieux M (1999) The role of preoperative chemotherapy in the treatment of nephroblastoma: the SIOP experience. *Semin Urol Oncol* **17**: 28–32

Green DM and Coppes MJ (1995) Future directions in clinical research in Wilms' tumor. *Hematol Oncol Clin North Am* **9**: 1329–1339

Green DM, Finklestein JZ, Norkool P and D'Angio GJ (1988) Severe hepatic toxicity after treatment with single-dose dactinomycin and vincristine. *Cancer* **62**: 270–273

Green DM, Breslow NE, Evans I, Moksness J, Finklestein JZ, Evans AE and D'Angio GJ (1995) Relationship between dose schedule and charges for treatment on national Wilms' tumor study 4. A report from the National Wilms' Tumor Study Group. *J Natl Cancer Inst* **19**: 21–25

Green DM, D'Angio GJ, Beckwith JB, Breslow NE, Grundy PE, Ritchey ML and Thomas PR (1996*a*) Wilms' Tumor. *CA Cancer J Clin* **46**: 46–63

Green DM, Breslow NE, Evans I, Moksness J and D'Angio GJ (1996*b*) Treatment of children with stage IV favorable histology Wilms' tumor: a report from the National Wilms' Tumor Study Group. *Med Pediatr Oncol* **6**: 147–152

Green DM, Breslow NE, Beckwith JB, Finklestein JZ, Grundy P, Thomas PR, Kim T, Shochat S, Haase G, Ritchey M, Kelalis P and D'Angio GJ (1998) Effect of duration of treatment on treatment outcome and cost of treatment for Wilms' tumour: a report from the National Wilms' Tumour Study Group. *J Clin Oncol* (United States) **16**: 3744–3751

Kaplan EL and Meier P (1958) Non-parametric estimation from incomplete observations. *Journal of the American Statistical Association* **53**: 457–481

Kumar R, Fitzgerald R and Breatnach F (1998) Conservative management of bilateral Wilms' tumor: results of the United Kingdom Children's Cancer Study Group. *J Urol* **160**: 1450–1453

Lennox EL, Stiller CA, Morris-Jones PH and Kinnier-Wilson LM (1979) Nephroblastoma: treatment during 1970–73 and the effect on survival of inclusion in the first MRC trial. *BMJ* **2**: 567–569

Lipshultz SE, Colan SD, Gelber RD Perez-Attayde AR, Sallan SE and Sanders SP (1991) Late cardiac effects of doxorubicin therapy for acute lymphoblastic leukemia in children. *New Engl J Med* **324**: 808–815

Ludwig R, Weirich A, Hofman WJ, and Waldherr R (1992) Veno-occlusive disease as a hepatotoxic side-effect of the Nephroblastoma SIOP-9 protocol: preliminary results of the German Group. *Med Pediatr Oncol* **20**: 434

Morris-Jones P, Marsden HB, Pearson D and Barnes J (1987) MRC second nephroblastoma trial, 1974–78: long-term results. *SIOP Proceedings 1987* Abstr. 121. SIOP: Jerusalem

Pachnis A, Pritchard J, Gaze M, Levitt G and Michalski AM (1998) Radiotherapy omitted in the treatment of selected children under 3 years of age with stage III favorable histology Wilms' tumor. *Med Pediatr Oncol* **31**: 150–152

Peto R, Pike C, Armitage P, Breslow NE, Cox DR, Howard SV, Mantel N, McPherson K, Peto J and Smith PG (1977) Design and analysis of randomized clinical trials requiring prolonged observation of each patient. *Br J Cancer* **35**: 1–37

Pritchard J, Imeson J, Barnes J, Cotterill S, Gough D, Marsden HB, Morris-Jones P and Pearson D (1995) Results of the United Kingdom Children's Cancer Study Group (UKCCSG) First Wilms' Tumor Study (UKW-1). *J Clin Oncol* **13**: 124–33

Raine J, Bowman A, Wallendszus K and Pritchard J (1991) Hepatopathy-thrombocytopenia syndrome. A complication of actinomycin-D therapy for Wilms' tumours. *J Clin Oncol* **9**: 268–273

Sorensen K, Levitt G, Sebag-Montefiore D, Bull C and Sullivan I (1995) Cardiac function in Wilms' tumour survivors. *J Clin Oncol* **13**: 1546–1556

Tournade MF, Com-Nogue C, Voute PA, Lemerle J, de Kraker J, Delemarre, Burgers M, Habrand JL, Moorman CGM, Burger D, Rey A, Zucker JM, Carli M, Jereb B, Bey P, Gauthier F and Sandstedt B (1993) Results of the sixth International Society of Paediatric Oncology Wilms' tumor trial and study: a risk adapted approach in Wilms' tumor. *J Clin Oncol* **11**: 1014–1023

*© 2000 Cancer Research Campaign*

### APPENDIX

### Primary investigators contributing to UKW1

Dr Derek King, Aberdeen
Dr Sid Dempsey, Belfast
Dr Jill Mann, Birmingham
Dr Martin Mott, Bristol
Dr Valerie Broadbent, Cambridge
Dr Eileen Thompson, Cardiff
Dr Fin Breatnach, Dublin
Dr Tim Eden, Edinburgh
Dr Ann Barrett, Glasgow
Dr Cliff Bailey, Leeds
Dr Rosemary Shannon, Leicester
Dr John Martin, Liverpool
Dr Jon Pritchard, London
Dr Judith Kingston, London
Dr Pat Morris Jones, Manchester
Dr Alan Craft, Newcastle
Dr Peter Barbor, Nottingham
Dr John Lilleyman, Sheffield
Dr Martin Radford, Southampton
Dr Ross Pinkerton, Sutton

*© 2000 Cancer Research Campaign*
